# Molecular Alterations in the Stomach of *Tff1*-Deficient Mice: Early Steps in Antral Carcinogenesis

**DOI:** 10.3390/ijms21020644

**Published:** 2020-01-18

**Authors:** Eva B. Znalesniak, Franz Salm, Werner Hoffmann

**Affiliations:** Institute of Molecular Biology and Medicinal Chemistry, Otto-von-Guericke University Magdeburg, Leipziger Str. 44, 39120 Magdeburg, Germany; eva.znalesniak@med.ovgu.de (E.B.Z.); franz.salm@med.ovgu.de (F.S.)

**Keywords:** trefoil factor, TFF1, TFF2, FCGBP, gastric cancer, carcinogenesis, oxidative stress, ROS, inflammation, innate immunity

## Abstract

TFF1 is a peptide of the gastric mucosa co-secreted with the mucin MUC5AC. It plays a key role in gastric mucosal protection and repair. *Tff1*-deficient (*Tff1^KO^*) mice obligatorily develop antropyloric adenoma and about 30% progress to carcinomas. Thus, these mice represent a model for gastric tumorigenesis. Here, we compared the expression of selected genes in *Tff1^KO^* mice and the corresponding wild-type animals (RT-PCR analyses). Furthermore, we systematically investigated the different molecular forms of Tff1 and its heterodimer partner gastrokine-2 (Gkn2) in the stomach (Western blot analyses). As a hallmark, a large portion of murine Tff1 occurs in a monomeric form. This is unexpected because of its odd number of seven cysteine residues. Probably the three conserved acid amino acid residues (EEE) flanking the 7th cysteine residue allow monomeric secretion. As a consequence, the free thiol of monomeric Tff1 could have a protective scavenger function, e.g., for reactive oxygen/nitrogen species. Furthermore, a minor subset of Tff1 forms a disulfide-linked heterodimer with IgG Fc binding protein (Fcgbp). Of special note, in *Tff1^KO^* animals a homodimeric form of Gkn2 was observed. In addition, *Tff1^KO^* animals showed strongly reduced *Tff2* transcript and protein levels, which might explain their increased sensitivity to *Helicobacter pylori* infection.

## 1. Introduction

TFF1, a member of the trefoil factor family (TFF), is a typical secretory peptide of gastric surface mucous cells, where it is synthesized together with the mucin MUC5AC [1,2,3,4,5]. Human TFF1 consists of 60 amino acid residues including seven cysteine residues. This odd number allows disulfide-linked heterodimerization with gastrokine-2 (GKN2), which is also a secretory product of surface mucous cells [6,7]. In the human stomach, also relatively low amounts of TFF1 monomers and homodimers were described [8]. The TFF1 content in gastric juice has been reported to 70 ng/mL [1]. Originally, TFF1 (previous name: pS2) was discovered in breast cancer cells and it is considered as a positive prognostic factor [3]. TFF1 is ectopically expressed in a variety of tumors as well as in inflammatory disorders [3,4]. Generally, TFF1, like the other TFF peptides, appears to play an important role in mucosal protection and repair, and thus has therapeutic potential and clinical perspectives [9].

Inactivation of *Tff1* in mice (*Tff1^KO^*) clearly revealed a characteristic gastric phenotype [10]. All *Tff1^KO^* mice developed antropyloric adenoma and about 30% progressed to carcinomas [10]. Thus, these mice represent a recognized model for gastric tumorigenesis because the loss of TFF1 expression is one of the most frequent molecular alterations in human gastric carcinogenesis [11,12,13]. The multi-step carcinogenesis cascade is associated with NF-κB-mediated inflammation [14]. For example, the pro-inflammatory genes *Cxcl1* and *Cxcl5* were upregulated [14]. The selective Cox-2 inhibitor celecoxib strongly reduced the development of gastric dysplasia and induced ulceration and inflammation of the adenoma suppressing tumor growth [11,14,15]. Thus, TFF1 appears to function as a gastric tumor suppressor [11]. This is in line with reports that *Tff1^KO^* mice are particularly susceptible to chemically induced tumorigenesis [16,17]. However, the precise molecular function of TFF1 as a tumor suppressor has not been elucidated thus far.

Furthermore, TFF1 gain- and loss-of-function experiments showed that TFF1 promotes cell migration and invasion of cells indicating that TFF1 is a motogenic (chemotactic) factor [3,4,16,18,19]. This motogenic effect is supported by an anti-apoptotic function [20]. Thus, both activities would be well suited to synergistically enhance rapid repair of damaged mucous epithelia by restitution [21,22]. For example, in different animal models of intestinal damage, Tff1 appeared to have a protective role either when overexpressed in transgenic mice or when delivered by genetically modified *Lactococcus lactis* [23,24]. Furthermore, a formulation of *L. lactis* secreting TFF1 has also been used to treat radiation-induced oral mucositis in an animal model [25]. This is a promising step towards further clinical applications, e.g., for treating cancer patients receiving chemotherapy [9]. However, the motogenic effects of TFF peptides generally appear to be rather weak (effective in a concentration range of about 10^−6^ to 10^−7^ M; [18]).

Generally, in *Tff1^KO^* animals the differentiation of the gastric mucosa from stem and precursor cells is dysregulated [11]. Antral pits are hypertrophic and these cells are almost devoid of mucus [10,11]. Additionally, the differentiation of fundic pit and parietal cells as well as antral gland cells is dysregulated [26,27].

In the past, homodimeric TFF1 has also been demonstrated to act as a lectin, e.g., it interacts with a lipopolysaccharide of *Helicobacter pylori* in a pH-dependent manner [28]. Thus, TFF1 may have a role in mediating the tropism of *H. pylori* within the gastric mucus [29].

Here, we compared the expression of selected genes in *Tff1^KO^* mice and wild-type animals at the age of 20 weeks. At that time, the first foci of carcinoma develop in about 30% of the *Tff1^KO^* mice [10]. Furthermore, for the first time, we systematically investigated the different molecular forms of Tff1 and its heterodimeric binding partner Gkn2. In addition, also Tff2 and Fcgbp were examined on the protein level. This is a further step in order to understand the molecular function of Tff1, in particular as a tumor suppressor.

## 2. Results

### 2.1. Transcriptional Alterations in Tff1^KO^ and Wild-Type Animals (RT-PCR Analyses)

**Figure 1:**
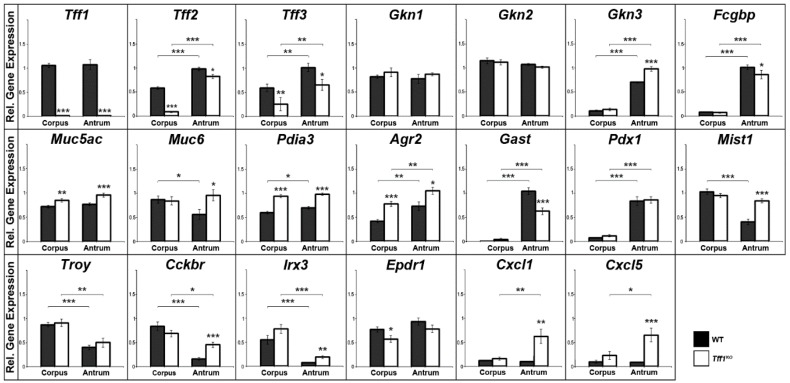
Semi-quantitative RT-PCR analyses. *Tff1* (21x), *Tff2* (20x), *Tff3* (30x), *Gkn1* (21x), *Gkn2* (22x), *Gkn3* (24x), *Fcgbp* (31x), *Muc5ac* (25x), *Muc6* (27x), *Pdia3* (25x), *Agr2* (24x), *Gast* (26x), *Pdx1* (33x), *Mist1* (35x), *Troy* (35x), *Cckbr* (33x), *Irx3* (35x), *Epdr1* (33x), *Cxcl1* (32x), and *Cxcl5* (32x) expression was monitored in the corpus and antrum of 10 male wild-type (WT) and 10 male *Tff1^KO^* mice. The relative gene expression levels were normalized against β-actin (*Actb*, 24x). The number of amplification cycles is given in parentheses. Significances are indicated by asterisks (*, *p* ≤ 0.05; **, *p* ≤ 0.01; ***, *p* ≤ 0.001). WT animals: black bars; *Tff1^KO^* animals: white bars.

Relative gene expression levels were investigated in the gastric corpus and antrum of *Tff1^KO^* and wild-type mice. Figure 1 represents mainly a selection of genes, which showed significant differences between wild-type and *Tff1^KO^* mice or between the corpus and the antrum. For example, the expression of genes was monitored, which encode typical secretory proteins of gastric mucous cells, such as *Tff1-3*, gastrokines (*Gkn1-3*), *Fcgbp*, mucins (*Muc5ac*, *Muc6*), and disulfide isomerases (*Pdia3*, *Agr2*). Additionally, markers for the gastric antrum (gastrin: *Gast*; transcription factor *Pdx1*), a marker for the differentiation of the mucous neck cell-zymogenic cell lineage (*Mist1*), the stem cell markers *Troy* and gastrin CCK-B receptor (*Cckbr*), and a marker for the gastric corpus (*Irx3*) are shown. Furthermore, expression of the ependymin related protein 1 (*Epdr1*) was monitored. For comparison and as controls, *Cxcl1* and *Cxcl5* were also included [14].

As a hallmark, *Tff1^KO^* animals showed a reduced *Tff2* expression particularly in the corpus, when compared with the wild-type animals. Additionally, *Tff3* expression is significantly reduced in *Tff1^KO^* animals. Both, *Gkn3* and *Fcgbp* expression are significantly higher in the antrum when compared with the corpus; in the antrum of *Tff1^KO^* animals, *Gkn3* is significantly elevated, whereas *Fcgbp* is significantly reduced when compared with the wild-type. Furthermore, the transcript levels of both disulfide isomerase genes *Pdia3* and *Agr2* were significantly higher in the *Tff1^KO^* animals. In contrast, gastrin (*Gast*) expression was significantly downregulated in the antrum of *Tff1^KO^* mice. Of note, the expression of *Mist1*, *Cckbr*, *Irx3* as well as that of *Cxcl1* and *Cxcl5* was significantly elevated in the antrum of *Tff1^KO^* animals. *Epdr1* expression was significantly reduced in the corpus of *Tff1^KO^* animals.

### 2.2. Tff1^KO^ and Wild-Type Animals Differ in Their Gkn2 Protein Forms (Western Blot Analyses)

**Figure 2:**
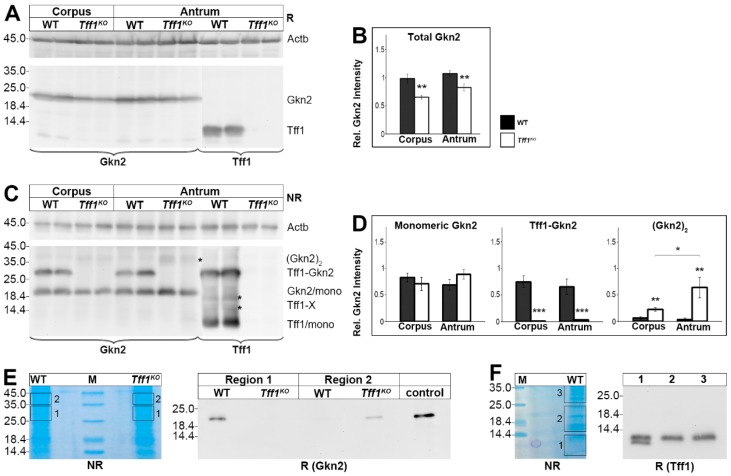
Western blot analyses of gastric corpus and antrum extracts of wild-type (WT) and *Tff1^KO^* animals. (**A**) Extracts of two male animals of each group were separated on a 15% SDS-PAGE under reducing conditions, and analyzed for Gkn2 and Tff1, respectively. Left: molecular mass standard. Loading control: reactivity for ß-actin (Actb). (**B**) Semi-quantitative analysis of the relative Gkn2 content (10 male animals per group). (**C**) Analysis of the same samples as in (A), but under non-reducing conditions. Marked are the positions of the Gkn2 homodimer ((Gkn2)_2_; indicated by an asterisk), the Tff1-Gkn2 heterodimer, the Gkn2 monomer, possible Tff1-X homo/heterodimers (indicated by asterisks), and the Tff1 monomer. (**D**) Semi-quantitative analysis of relative monomeric Gkn2, heterodimeric Tff1-Gkn2, and homodimeric (Gkn2)_2_ contents, respectively (10 male animals per group). (**E**) Left: Non-reducing SDS-PAGE (Coomassie-stained) of antral extracts pooled from three female WT and three female *Tff1^KO^* animals (55 µg protein per lane) and elution of the regions 1 and 2; M, molecular mass standard. Right: Reducing Western blot analysis of the eluates concerning Gkn2. (**F**) Left: Non-reducing SDS-PAGE (Coomassie-stained) of a corpus extract of a single male WT mouse and elution of the regions 1–3. Right: Reducing Western blot analysis of the eluates concerning Tff1. Significances are indicated by asterisks (*, *p* ≤ 0.05; **, *p* ≤ 0.01; ***, *p* ≤ 0.001). WT animals: black bars; *Tff1^KO^* animals: white bars.

Since human TFF1 has been reported to form a heterodimer with GKN2 [6,7], we analyzed the Tff1 and Gkn2 protein levels in more detail. Clearly, Tff1 was present in wild-type animals only; whereas Gkn2 was detectable in both wild-type and *Tff1^KO^* animals (Figure 2A). A semi-quantitative analysis showed that the total Gkn2 level is significantly lowered in both the corpus and the antrum of *Tff1^KO^* animals (Figure 2B).

Under non-reducing conditions, both monomeric Tff1 as well as monomeric Gkn2 were detected (Figure 2C). The amount of monomeric Gkn2 is comparable in wild-type and *Tff1^KO^* animals (Figure 2D). Furthermore, also the Tff1-Gkn2 heterodimer was clearly detectable in wild-type animals with both antisera against Tff1 and Gkn2, respectively (Figure 2C). As expected, the Tff1-Gkn2 heterodimer is missing in *Tff1^KO^* animals (Figure 2C,D). In return, in the *Tff1^KO^* animals a weak 37k-Gkn2-band was detected, particularly in the antrum (indicated by an asterisk in Figure 2C). This band is expected to represent homodimeric Gkn2 and is significantly elevated in both the corpus and antrum of *Tff1^KO^* animals (Figure 2D).

The specificity of this 37k-band was confirmed by elution from a non-reducing gel (region 2) followed by Western blot analysis after reducing SDS-PAGE (Figure 2E). Clearly, the 37k-band was shifted to monomeric Gkn2 in the *Tff1^KO^* animals only (Figure 2E). For comparison, also region 1 containing the Tff1-Gkn2 heterodimer was eluted and analyzed under reducing conditions. As expected, Tff1-Gkn2 was shifted to monomeric Gkn2 in the wild-type animals only (Figure 2E). Thus, *Tff1^KO^* and wild-type animals differ characteristically in their Gkn2 forms.

Under non-reducing conditions, multiple forms of Tff1 were detected in wild-type animals, i.e., the Tff1-Gkn2 heterodimer, the monomeric Tff1, and at least two weak bands between (16-20k region; Tff1-X; indicated by asterisks in Figure 2C). In order to test if the signals in the 16-20k region are specific, this region (region 2) as well as monomeric Tff1 (region 1) and Tff1-Gkn2 (region 3) were eluted from a non-reducing gel followed by Western blot analysis under reducing conditions (Figure 2F). Clearly, monomeric Tff1 was detected in all three regions analyzed indicating that also the 16-20k region contained a dimeric Tff1 form. In addition, region 1 also contained a shortened Tff1 form.

### 2.3. Tff1 Is Capable of Forming a Heteromer with Fcgbp, Particularly in the Gastric Antrum

In the past, in both the human intestine and saliva TFF3 has been found to form a disulfide-linked heterodimer with FCGBP [30,31]. As TFF1 and FCGBP are both secretory products of gastric surface mucous cells and the overall structure of TFF1 and TFF3 is very similar including the occurrence of seven cysteine residues, we tested whether Tff1 forms a complex with Fcgbp in the murine stomach.

**Figure 3:**
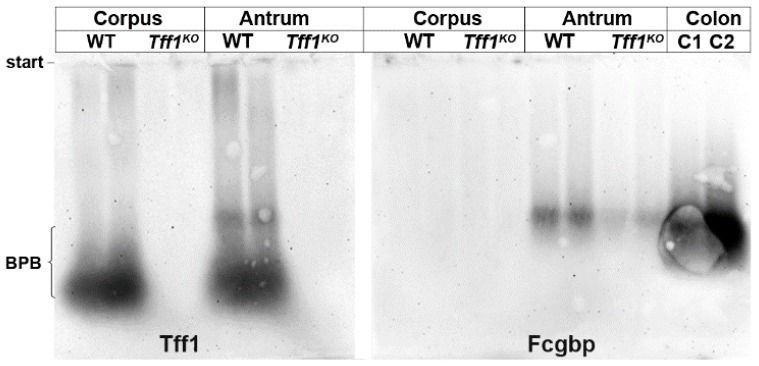
Western blot analyses of gastric corpus and antrum extracts of female wild-type (WT) and female *Tff1^KO^* animals. Extracts of two animals per group were separated by 1% agarose gel electrophoresis. Shown are the reactivities for Tff1 and Fcgbp, respectively. As a positive control for Fcgbp, two murine colon extracts were analyzed (C1, C2). The start and the dye bromophenol blue (BPB) are marked on the left.

As shown in Figure 3, in the murine antrum little Tff1 was traceable also in a high-molecular-mass form; whereas the bulk of Tff1 mainly appeared in the low-molecular-mass region. This high-molecular-mass band was detectable in the same region as Fcgbp, which was again present in the antrum only. As a control, Fcgbp was demonstrated in the colon with a comparable molecular mass. This indicates that part of gastric Tff1 forms a heteromer with Fcgbp. Furthermore, the Fcgbp level was reduced in the antrum of *Tff1^KO^* animals when compared with the wild-type.

### 2.4. Tff1^KO^ Mice Show Strongly Reduced Tff2 Levels

**Figure 4:**
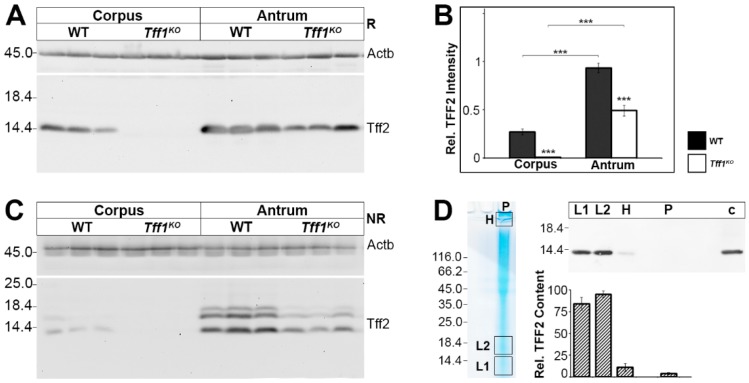
Western blot analyses of gastric corpus and antrum extracts of wild-type (WT) and *Tff1^KO^* animals. (**A**) Extracts of three male animals per group were separated on a 15% SDS-PAGE under reducing conditions and analyzed for their Tff2 reactivity. Left: molecular mass standard. Loading control: reactivity for ß-actin (Actb). (**B**) Semi-quantitative analysis of the relative Tff2 content (10 male animals per group). Significances are indicated by asterisks (***, *p* ≤ 0.001). WT animals: black bars; *Tff1^KO^* animals: white bars. (**C**) Analysis of the same samples as in (A), but under non-reducing conditions (post-in-gel reduction). (**D**) Left: Non-reducing SDS-PAGE (Coomassie-stained) of an antral extract of a single male WT animal (75 µg) followed by elution of the high-(H) and low-molecular mass regions (L1, L2). Additionally, the remaining high-molecular-mass samples not entering the gel were removed from the gel pockets (P). Right: Western blot analysis (reducing conditions) concerning Tff2 of L1, L2, H, P, and an antral extract as a control (c) and semi-quantitative analysis of the relative Tff2 content (six male WT animals; 60–75 µg/lane).

As the *Tff2* transcript level is diminished in *Tff1^KO^* animals, particularly in the corpus (Figure 1), we analyzed the Tff2 content also at the protein level (Figure 4). Western blot analysis clearly revealed that in wild-type animals the Tff2 level is much higher in the antrum when compared with the corpus (Figure 4A,B). Furthermore, in *Tff1^KO^* animals the Tff2 protein level was significantly lower when compared with the wild-type animals (Figure 4B).

Of note, under non-reducing conditions Tff2 appeared as three bands, i.e., the regular 14k monomeric band and two additional bands (about 16k, 17k; Figure 4C). The specificity of the 16k/17k-double band (region L2) was confirmed by elution from a non-reducing gel followed by Western blot analysis after reducing SDS-PAGE (Figure 4D). As a control, also the regular 14k band (region L1) was analyzed accordingly. Clearly, the 16k/17K-double band (region L2) shifted to monomeric Tff2 indicating that these entities represent indeed other forms of Tff2. The Tff2 content in both regions L1 and L2 is comparable (Figure 4D). In addition, the Tff2 content of the high-molecular-mass region as well as the gel pocket was determined; both did not contain significant amounts of Tff2 (Figure 4D).

## 3. Discussion

### 3.1. Transcriptional Alterations in Tff1^KO^ Animals Are Indicative for Pre-Neoplastic Changes in the Antrum

The gastric mucosae in the corpus and antrum differ not only in their architecture, but also their continuous self-renewal occurs from different sets of stem cells [32]. Expression profiling with the gastric antral markers *Pdx1* and *Gast* clearly revealed that corpus and antrum samples were collected properly. In contrast, *Troy* expression is significantly higher in the corpus, which is in line with the observation that *Troy* stem cells are located at the base of fundic units [32]. Of special note, gastrin expression in the antrum of *Tff1^KO^* mice is significantly reduced. This agrees with a recent report that gastrin is among the top downregulated genes in antral low-grade dysplasia in *Tff1^KO^* animals [13]. Thus, this downregulation of gastrin might be an additional factor supporting antral carcinogenesis because *Gast^KO^* mice are known to develop antral tumors; in contrast, increased gastrin levels promote corpus carcinogenesis [33].

*Gkn3*, in contrast to *Gkn1* and *Gkn2*, is mainly expressed in the antrum. This is in line with a previous report [34]. *Gkn3* is known to be mainly co-expressed with *Tff2* and *Muc6* in antral glands, and little *Gkn3* is expressed in a subpopulation of mucus neck cells in the corpus [34,35]. In *Tff1^KO^* animals, *Gkn3* expression is significantly upregulated in the antrum; whereas *Gkn1* and *Gkn2* expression is not altered in these animals. This might be a sign for beginning tumorigenesis because a characteristic *Gkn3* upregulation has been reported in a murine antral tumorigenesis model (*gp130^F/F^*) [35]. Thus, the specific upregulation of *Gkn3* expression in the antrum of *Tff1^KO^* mice could reflect the amplification of the proliferating gland progenitor cells observed in these animals [27]. These proliferating progenitors probably become the invasive cells of the gastric adenocarcinomas [27].

Expression of *Cxcl1* and *Cxcl5* is significantly increased in the antrum of *Tff1^KO^* animals, but not in the corpus. This is in agreement with a previous report indicating that loss of *Tff1* is associated with activation of NF-κB-mediated inflammation [14].

Furthermore, expression of the transcription factor *Mist1* is significantly increased in the antrum of *Tff1^KO^* animals. This is most interesting because *Mist1* is a marker for the differentiation of chief cells and for a discrete population of stem cells in fundic units on the one hand and long-lived precursor cells in antral units on the other hand [36]. The latter are located at the position +5 within a Cxcl12(Sdf1)/Cxcr4 perivascular niche and they have been shown to be capable of acting as an origin of antral tumors [36]. Thus, the increased expression of *Mist1* in *Tff1^KO^* animals specifically in the antrum might be a sign for the expansion of these precursor cells, which is an early step during antral carcinogenesis [36]. Deletion of *Cxcl12* in endothelial cells or pharmacological blockage of Cxcr4 is known to inhibit antral tumor growth [36]. Thus, it would be highly interesting to test whether AMD3100, a specific inhibitor of CXCR4, would also reduce the number and/or size of the antral tumors in *Tff1^KO^* animals.

Expression of the gastrin receptor *Cckbr* mainly occurs in enterochromaffine-like (ECL) and parietal cells of the gastric corpus, but also in a long-lived stem cell population at position +4 in the gastric antrum [37]. The significantly upregulated expression of *Cckbr* specifically in the antrum of *Tff1^KO^* animals might be an indication for first neoplastic changes.

*Irx3* (together with *Irx2* and *Irx5*) has been described as a transcription factor typical of the embryonic gastric fundus [38]. Thus, the predominant expression in the corpus of wild-type animals might be expected. However, the significantly upregulated expression of *Irx3* in the antrum of *Tff1^KO^* animals is surprising and could be a sign for neoplastic changes.

Additionally, expression of ependymin-related protein 1 (*Epdr1*; previously termed *Merp2*) was monitored, because it was downregulated in a murine asthma model; whereas *Tff1* was upregulated in this model [39]. Thus, it was tested whether *Epdr1* expression was related to an inflammatory process in the stomach of *Tff1^KO^* mice. However, in *Tff1^KO^* animals *Epdr1* is significantly downregulated in the corpus only. The reason for this is not clear currently because NF-κB-mediated inflammation is detectable in the antrum, but not the corpus, as shown by increased *Cxcl1* and *Cxcl5* expression.

### 3.2. A Large Portion of Murine Tff1 Occurs in a Monomeric Form: A Possible Protective Role

In wild-type animals, a relatively large portion of Tff1 is present as a monomer (Figure 2C). In contrast, a homodimeric form of Tff1 (expected to be in the 18–24k range [30]) is hardly detectable (asterisk in Figure 2C). Thus, we eluted the corresponding region 2 (16–24k) and could indeed detect significant amounts of Tff1 (Figure 2F). This is indicative that in region 2 probably homodimeric Tff1 is present and maybe also another heterodimeric Tff1-X form with an unknown partner protein (the two bands are indicated by asterisks in Figure 2C). Furthermore, when monomeric Tff1 (region 1) was eluted and analyzed again, a double band was detected (Figure 2F). Thus, Tff1 also exists in a shortened form maybe similar as shown for human TFF3 recently [31]. This shortened form is also visible in Figure 2A. Theoretically, this form could originate from alternative cleavage sites by signal peptidase. From cDNA cloning, an unusual N-terminal sequence was predicted for Tff1 (QAQAQAQAQEE), which would allow multiple cleavages by signal peptidase and cyclisation of an N-terminal Gln residue [2].

The robust occurrence of monomeric Tff1 is unusual because Tff1 contains an odd number of seven cysteine residues, which should form disulfide-linked homo- or heterodimers. Generally, the oxidation machinery of the endoplasmic reticulum enforces disulfide bond formation [40]; whereas exposed unpaired thiols act as intracellular retention signals for unassembled secretory proteins, which are then subject to degradation [41]. However, there are proteins known, which are secreted in spite of an unpaired cysteine residue, such as Ig light chains [41]. Here, a flanking acidic residue masks the retention signal and allows transport to the Golgi [41]. Such a case probably also occurs in murine Tff1 where the C-terminal 7th Cys residue is flanked upstream by even three glutamic acid residues [2]. The negative charges of the flanking glutamic acid residues might be the reason why probably little homodimerization of Tff1 was observed (Figure 2C). Furthermore, also human TFF1 when produced in *Pichia pastoris* was secreted as a monomer [42] as well as the *Xenopus laevis* ortholog of TFF1, i.e., xP1, occurs as a monomer [43]. In both, human TFF1 and frog xP1 the 7th cysteine residue is flanked by four or a single glutamic acid residue, respectively [44,45]. Thus, the occurrence of acid amino acid residues flanking the 7th cysteine residue and the secretion as a monomer seem to be evolutionary conserved features of TFF1/Tff1/xP1. These flanking acid residues would also explain why the intermolecular disulfide bridge in human recombinant TFF1 dimer (through Cys-58) is more susceptible to reduction than the intramolecular disulfide bridges [46]. Of note, the cluster of acid amino acid residues was reported to bind Cu^2+^ in vitro [47].

The question arises on the molecular function of this unpaired 7th cysteine residue in monomeric Tff1. A plausible hypothesis would be that this 7th cysteine residue could have a scavenger function, e.g., for extracellular reactive oxygen/nitrogen species (ROS/RNS). Generally, such modifications preferentially occur on a subset of reactive cysteine residues [48]. Activation of these cysteine residues occurs via flanking amino acid residues, which change the pKa favoring the existence of thiolate anions [48,49,50]. The cluster of three flanking acid residues is highly unusual and is present only in TFF1/Tff1 [3]. The apical surface of gastric epithelial cells is well known to be target for great oxidative stress because, e.g., of the release of extracellular ROS by dual oxidase (DUOX) particularly during bacterial infections and chronic inflammatory diseases [51,52]. Thus, effective protection of the gastric surface is essential to prevent stomach disorders [53]. Monomeric Tff1 would be perfectly designed to fulfill such a protective function and its loss could well explain the occurrence of adenomas in the antrum of *Tff1^KO^* animals because the antrum is the preferred region for bacterial colonization, e.g., *H. pylori* [54]; whereas the fundic region is mainly protected from infection by its acidic pH.

For the future, it would be interesting to test the ROS/RNS scavenger hypothesis for Tff1 experimentally. For example, it could be investigated whether oral application of short synthetic peptides mimicking the activated 7th cysteine residue of Tff1 could prevent carcinogenesis in *Tff1^KO^* animals and whether this is superior when compared with *N*-acetylcysteine.

A ROS/RNS scavenger function of Tff1 would also explain why certain probiotics have beneficial effects on their hosts. For example, the probiotic mixture VSL#3 has been shown to raise the production of Tff1 and Tff2 in mice [55].

Furthermore, a role for Tff1 as a scavenger for ROS/RNS could also be the reason why Tff1 is ectopically expressed during various inflammatory conditions in mice, such as encephalitis, asthma, pancreatitis, and in the murine spleen after *Toxoplasma gondii* infection [39,56,57,58]. Here, the reactive thiol of Tff1 could protect the tissue from damage by the oxidative burst.

In addition, the unpaired 7th cysteine residue in Tff1 might also have a transient function for the correct intracellular folding of the mucin Muc5ac, which is synthesized and secreted by just the same cells. Such a function during the secretory pathway would explain why in antropyloric tumors of *Tff1^KO^* animals the unfolded protein response is activated [59]. Remarkably, the expression of the disulfide isomerases *Pdia3* and *Agr2* is significantly upregulated in *Tff1^KO^* animals (Figure 1), which might be a further indication for a folding function of Tff1 (and possibly also of Tff2).

### 3.3. Tff1 Forms a Complex with Fcgbp in the Antrum

Here, we report for the first time that minor amounts of Tff1 can serve as a heteropartner for Fcgbp (Figure 3). This result is in agreement with the observation that Fcgbp is mainly expressed in the antrum (Figure 1 and Figure 3). A similar observation has been reported for the human stomach, where FCGBP is mainly expressed in surface mucous cells of the antrum together with TFF1 [60].

Consequently, our results imply that the expression profiles of surface mucous cells in the murine corpus and antrum differ, just as has been documented for human [60]. One reason might be that the antrum is the preferred site for bacterial infection [54]. This is in line with the observation that FCGBP is the highest upregulated early defense gene in fish skin after microbial infection [61]. The formation of a Tff1-Fcgbp heterodimer in the gastric mucosa is not really surprising because the overall structures of Tff1 and Tff3 are similar; the latter is already known to form disulfide-linked TFF3-FCGBP heterodimers in the human intestine and saliva [30,31].

The molecular functions of Fcgbp and the Tff1-Fcgbp heterodimer are not understood thus far. Generally, FCGBP is considered as a component of the first line innate immune defense, e.g., it is a typical early response gene after microbial infection probably regulating pathogen attachment and disease progression at mucosal surfaces [61,62] including a viral trap for HIV-antibody complexes [63]. FCGBP is ubiquitous in vertebrates and cephalochordates [64]. Of note, even a number of bacterial proteins have N-terminal domains homologous to the N-terminal domain of FCGBP [64]. The modular structure of FCGBP (cysteine-rich domains) could well support a function for the clearing of microorganisms. This would also explain why Fcgbp is mainly expressed in the antrum (Figure 1 and Figure 3) because here bacterial colonization preferentially occurs [54]. The heterodimerization with Tff1 could synergistically support the binding of bacteria by Fcgbp, in particular by its known lectin activity [28]. Furthermore, FCGBP has also been reported to be part of a net-like scaffold within mucosal barriers [65].

### 3.4. Tff1^KO^ and Wild-Type Mice Synthesize Different Gkn2 Forms, Particularly in the Antrum

In wild-type animals, relatively large proportions of Gkn2 exist as a Tff1-Gkn2 heterodimer (Figure 2C) and the remaining Gkn2 appears as a monomer (Figure 2C). Heterodimeric interaction between TFF1 and GKN2 has been reported to entail synergistic anti-proliferative and pro-apoptotic effects on gastric cancer cells [66].

A hallmark of *Tff1^KO^* animals is the formation of a 37k-Gkn2 entity, probably a Gkn2 homodimer (asterisk in Figure 2C). This form is particularly present in the antrum and is nearly missing in wild-type animals (Figure 2D). The existence of this Gkn2 form is highly interesting because it is a primary consequence of the Tff1 deficiency. Generally, GKN2 is downregulated in gastric cancers and has anti-inflammatory and tumor suppressor roles [35,67,68]. Currently, the biological function of GKN2 is not known and it is also a matter of speculation how GKN2 exerts a biological activity. GKN2 might be a ligand for a receptor, either as a classical peptide ligand or a lectin. Such a hypothesis might be supported by a report that a synthetic peptide corresponding to the 21 C-terminal amino acid residues of the related peptide GKN1 has relatively weak mitogenic and motogenic properties [69]. Additionally, anti-proliferative activities have been reported for GKN1 [35]. Of note, GKN1 is not expected to form disulfide-linked dimers because it contains an even number of cysteine residues. Thus, it cannot be excluded that the 37k-form of Gkn2 has different biological effects when compared with the Tff1-Gkn2 heterodimer and this might influence the inflammatory process particularly in the antrum.

### 3.5. Tff1^KO^ Mice Show Strongly Reduced Tff2 Levels

In wild-type animals, the Tff2 protein level is much higher in the antrum when compared with the corpus (Figure 4B). This is in line with its expression in mucous neck and antral gland cells, the latter being more numerous. On the transcript level, the difference between corpus and antrum is also significant, but not that pronounced (Figure 1). A possible explanation could be that in the corpus *Tff2* transcripts are found in precursor cells, whereas in the antrum *Tff2* is transcribed in the mature cells [32,60]. This could lower the translational efficiency of *Tff2* in the corpus. A similar situation, that transcript and protein levels are not completely parallel, has also been observed for human gastric TFF3 [70]. Clearly, after non-reducing SDS-PAGE Tff2 appears as the expected monomer (about 14k-band), but also as an additional double band at about 16k/17k (Figure 4C). However, we did not detect clear signals indicating the existence of a potential Tff2 homodimer.

The specificity of the 16k/17k entities was confirmed by elution from a non-reducing gel and shift of these bands to the monomeric band after reducing SDS-PAGE (Figure 4D). A similar situation has been described recently for human TFF2 [71]. These bands could represent different circular Tff2 forms because Cys-6 and Cys-104 probably form a disulfide bridge similar as porcine TFF2 [72]. The disulfide bridge between Cys-6 and Cys-104 connecting the N- with the C-terminal end of porcine TFF2 has been shown to be particularly sensitive to reduction with glutathione [73]. Well conserved pairs of acidic residues flanking Cys-104 as well as Cys-78 and Cys-58 could be responsible for such isomerization reactions (intramolecular disulfide exchange reactions), because flanking acid amino acids are known to change the pKa of cysteine residues [49,50].

As a hallmark in *Tff1^KO^* animals, the Tff2 protein levels are significantly reduced when compared with the wild-type animals (Figure 4B). In addition, also the *Tff2* transcript levels are significantly downregulated in *Tff1^KO^* animals, particularly in the corpus (Figure 1). The Tff2 downregulation is in line with previous reports [10,26,74]. It could be a direct consequence of the disruption of the *Tff1* gene, because all three *Tff* genes are clustered on chromosome 17q and there are indications for coordinated expression of the *Tff* genes [75,76]. In addition, the reduced gastrin expression in *Tff1^KO^* mice (Figure 1) could also account for the reduced Tff2 expression because gastrin regulates the TFF2 promoter [77]. Tff2 is a lectin stabilizing the inner gastric mucus barrier layer keeping bacteria at a distance [71,78]. Thus, a reduction of the mucus barrier function by a reduced Tff2 level could favor permeation of microorganisms and chronic inflammation leading to adenomas in the antrum because this is the preferred region for microbial colonization in the stomach [54].

Furthermore, *Tff2* downregulation would also explain another phenotype of *Tff1^KO^* animals well, i.e., they react more sensitive to *H. pylori* infection [79]. About 30% of the *H. pylori*-infected *Tff1^KO^* animals developed invasive adenocarcinoma (and only 10% of uninfected *Tff1^KO^* mice), whereas wild-type mice did not develop any dysplastic or invasive gastric lesions [79]. The significantly reduced Tff2 protein level could allow permeation of the bacteria leading to chronic inflammation and carcinogenesis. Taken together, the loss of Tff1 and the accompanied reduction of Tff2 probably act synergistically favoring chronic inflammation and carcinogenesis in the antrum.

## 4. Materials and Methods

### 4.1. Animals

As described previously, a stable line has been established by back-crossing two male mice heterozygous for *Tff1* [10] (obtained from Dr. M.-C. Rio and Dr. C. Tomasetto, Institut de Génétique et de Biologie Moléculaire et Cellulaire [IGBMC], Illkirch, France) with 129/Svj mice [56]. Subsequently, this line was stabilized by back-crossing with C57BL/6J Rj mice (Janvier Labs, Saint-Berthevin Cedex, France) leading to a mixed background. Regions of the gastric corpus and antrum of 20 weeks old homozygous offspring (i.e., wild-type and *Tff1^KO^* animals) were investigated here.

### 4.2. DNA and RNA Extraction, PCR Analysis

Genotyping of *Tff1^KO^* mice has been reported in detail previously [80]. The primer pairs used for PCR analysis have been reported [56]. Isolation and purification of total gastric RNA (using TRIzol^TM^ Reagent; ambion by life technologies, Carlsbad/CA, USA) as well as RT-PCR analysis (reverse transcriptase: Takara Bio Europe, Saint Germain en Laye, France) and semi-quantitative evaluation of the relative expression levels of selected genes including statistical analysis was as described in detail previously [80]. Relative intensities were normalized against the corresponding relative β-actin intensities. Error bars represent ± SEM. 

The specific primer pairs used in this RT-PCR study have been published previously (*Epdr1*, MB1890/1891; *Fcgbp*, MB1516/1517; *Gkn3*, MB2656/2657; *Tff1*, MD7/MD8; *Tff3*, MB1847/MB1848; [56,58,80]) or are listed in Table 1 (*Actb*, *Agr2*, *Cckbr*. *Cxcl1*, *Cxcl5*, *Gast*, *Gkn1*, *Gkn2*, *Irx3*, *Mist1/Bhlha15*, *Muc5ac*, *Muc6*, *Pdia3*, *Pdx1*, *Tff2*, *Troy/Tnfrsf19*).

### 4.3. SDS-PAGE, Agarose Gel Electrophoresis, Western Blot Analysis, Antisera

Proteins were extracted using TRIzol^TM^ Reagent (ambion by life technologies, Carlsbad/CA, USA). The pellets were resuspended in sterile Milli-Q water containing 1% SDS, and a protease inhibitor mix (cOmplete^TM^, EDTA-free; Roche, Penzberg, Germany). The protein concentration was determined using the Pierce^TM^ BCA protein assay kit (Thermo Scientific^TM^, Rockford/IL, USA) according to the manufacturer’s instructions. For gel electrophoresis, 30 µg protein was loaded per lane except otherwise indicated.

Denaturing SDS-PAGE under reducing or non-reducing conditions, non-reducing agarose gel electrophoresis, and Western blot analysis have been described in detail previously [7,30,81]. Gels after non-reducing SDS-PAGE analyzed for Tff2 immunoreactivity were subjected to post-in-gel reduction with 1% mercaptoethanol at 50 °C for 2 min before blotting as described previously [82] followed by washing with Milli-Q water.

Murine Tff1 and Tff2 were detected with the affinity-purified polyclonal antisera anti-mTff1-1 (1:2000 dilution) [19] and anti-hTFF2-2 (1:1000 dilution), respectively; the latter being generated against the same antigen as anti-hTFF2-1 [83]. For detection of murine Gkn2, the polyclonal antiserum anti-hGKN2-1 was used (1:3000 dilution) [7]. Fcgbp was detected with a polyclonal antiserum (1:1000 dilution) against a 115k fragment of rat Fcgbp kindly provided by Prof. Jürgen Seitz (Philipps University, Marburg, Germany) [84]. Reactivity with a polyclonal anti-β-actin antiserum (1:500 dilution; code STJ97713, St. John’s Laboratory, London, UK) was used as loading control. 

Bands were visualized with the enhanced chemiluminescence (ECL) detection system (using a secondary antibody coupled to horseradish peroxidase and luminol/p-Coumaric acid/H_2_O_2_) and the intensity of the signals was semi-quantitatively analyzed as reported in detail previously [71]. The semi-quantitative analysis of the reactivity for Gkn2 and Tff2, respectively, was normalized after staining the blots with Amidoblack (Roth, Karlsruhe, Germany); here, the optical density was measured in equally sized boxes around the immunoreactive bands.

## 5. Conclusions

In wild-type animals, Tff1 occurs to large extent as a monomer. This is unusual and the unpaired thiol group of Tff1 might have a scavenger function for ROS reducing oxidative stress. Furthermore, relatively large amounts of Tff1-Gkn2 heterodimer were detected in the corpus and antrum of wild-type mice. In the antrum, little Tff1 also forms a heteromer with Fcgbp, analogous to human TFF3. There are indications that Tff1-Fcgbp has probably a function for clearing microorganisms, particularly in the antrum.

As a direct consequence of Tff1 deficiency, in *Tff1^KO^* animals a significant increase in a Gkn2 form was observed particularly in the antrum, which is likely homodimeric Gkn2. Currently, the biological function of this Gkn2 form is not known. Maybe, this Gkn2 entity has mitogenic or motogenic activities by analogy with Gkn1. Furthermore, *Tff2* transcript and protein levels are strongly downregulated. This weakens the inner gastric mucus barrier and makes it presumably more permeable for microorganisms.

Both the lack of protective Tff1 and the weakened gastric mucus barrier by downregulation of Tff2 are probably the major drivers for chronic damage of the gastric mucosa particularly in the antrum, because here microbial colonization occurs preferentially. As a secondary effect, chronic inflammation develops, which is then the base for carcinogenesis.

## Figures and Tables

**Table 1:** Oligonucleotides used for RT-PCR analysis and calculated size of the products.

Genes	Accession No.	Primer No.	Primer Pairs	Nucleotide Positions	Tm	Size (bp)	Intron Spanning
Actb	NM_007393.5	MB2658 MB2659	CACTGTCGAGTCGCGTCCA TGACCCATTCCCACCATCAC	29–47 255–236	60 °C	227	yes
Agr2	NM_011783.2	MB2778 MB2779	ACGAATGCCCACACAGTCAA GCGTAGAGCCGGTTTGAGTA	288–307 504–485	60 °C	217	yes
Cckbr	NM_007627.5	MB2674 MB2675	GCTGAGTGGGACTTCACAGG TTGGTGACCGTTCTTAGGCG	271–290 563–544	60 °C	293	yes
Cxcl1	NM_008176.3	MB2628 MB2629	GGCTGGGATTCACCTCAAGAA GTGTTGTCAGAAGCCAGCGT	183–203 420–401	60 °C	238	yes
Cxcl5	NM_009141.3	MB2636 MB2637	CAGTCATAGCCGCAACGGAG AGAAAATCCGTGGGTGGAGA	276–295 617–598	60 °C	342	yes
Gast	NM_010257.4	MB2450 MB2451	CGCCACAACAGCCAACTATT TTCGATGGTGAGAGGCTGAG	19–38 229–210	60 °C	211	yes
Gkn1	NM_025466.1	MB2454 MB2455	CCGCCATGAAGCTCACAATG CCAGGCCCTTTACCCTTCTG	39–58 372–353	60 °C	334	yes
Gkn2	NM_025467.1	MB2456 MB2457	AACATCCACTCAGGCTCGTG CTGTCGAATAGGCGACCCAA	185–204 454–435	60 °C	270	yes
Irx3	NM_001253822.1	MB2708 MB2709	AGCCGGAGAGTGGAACAGG GACATGCTTGCAACTCGTCAC	1799–1817 2041–2021	60 °C	243	yes
Mist1/ Bhlha15	NM_010800.4	MB2536 MB2537	GTGGCTAAAGCTACGTGTCC CTCCAGGCTGGTTTTCCCAG	8–27 122–103	60 °C	115	yes
Muc5ac	NM_010844.3	MB2700 MB2701	CCGCGTCAATGGAAAGTTGT CTGGAGGGTTGCATTGAGGT	3739–3758 4059–4040	60 °C	321	yes
Muc6	NM_001330001.2	MB2718 MB2719	GTTCCAGATGCAGCCTGTCT CATAGCTGAACGTGCGGTTG	1612–1631 2105–2086	60 °C	494	yes
Pdia3	NM_007952.2	MB2744 MB2745	CTAGTCGAGTTCTTCGCCCC AAAAACCCACCACTGAGGCA	282–301 615–596	60 °C	334	yes
Pdx1	NM_008814.4	MB2464 MB2465	ATCTCCCCATACGAAGTGCC GTTCCGCTGTGTAAGCACCT	335–354 572–553	60 °C	238	yes
Tff2	NM_009363.3	MB2306 MB2307	CTGGTAGAGGGCGAGAAACC TCTTGCGAGCTGACACTTCC	92–111 302–283	60 °C	211	yes
Troy/ Tnfrsf19	NM_013869.5	MB2684 MB2685	TCTCCTAGTTCGCCTGCCTT AAGAGCACCGTCCTGTGTAG	572–591 806–787	60 °C	235	yes

## Abbreviations

FCGBP	IgG Fc binding protein
SDS-PAGE	Sodium dodecyl sulfate-polyacrylamide gel electrophoresis
TFF	Trefoil factor family
